# Fine-Scale Habitat Segregation between Two Ecologically Similar Top Predators

**DOI:** 10.1371/journal.pone.0155626

**Published:** 2016-05-17

**Authors:** Francisco Palomares, Néstor Fernández, Severine Roques, Cuauhtemoc Chávez, Leandro Silveira, Claudia Keller, Begoña Adrados

**Affiliations:** 1 Department of Conservation Biology, Estación Biológica de Doñana, CSIC, Avda. Américo Vespucio s/n, 41092, Sevilla, Spain; 2 Universidad Autónoma Metropolitana-Unidad Lerma, Hidalgo Pte. 46, Col. La Estación, Lerma, Estado de México, 52006, México; 3 Jaguar Conservation Fund, Mineiros, Goias, Brazil; 4 Instituto Nacional de Pesquisas da Amazônia (INPA), Department of Biodiversity, CP 2223, 69065–970, Manaus, Amazonas, Brazil; University of Sydney, AUSTRALIA

## Abstract

Similar, coexisting species often segregate along the spatial ecological axis. Here, we examine if two top predators (jaguars and pumas) present different fine-scale habitat use in areas of coexistence, and discuss if the observed pattern can be explained by the risk of interference competition between them. Interference competition theory predicts that pumas should avoid habitats or areas used by jaguars (the dominant species), and as a consequence should present more variability of niche parameters across study areas. We used non-invasive genetic sampling of faeces in 12 different areas and sensor satellite fine-scale habitat indices to answer these questions. Meta-analysis confirmed differences in fine-scale habitat use between jaguars and pumas. Furthermore, average marginality of the realized niches of pumas was more variable than those of jaguars, and tolerance (a measure of niche breadth) was on average 2.2 times higher in pumas than in jaguars, as expected under the interference competition risk hypothesis. The use of sensor satellite fine-scale habitat indices allowed the detection of subtle differences in the environmental characteristics of the habitats used by these two similar top predators, which, as a rule, until now were recorded using the same general habitat types. The detection of fine spatial segregation between these two top predators was scale-dependent.

## Introduction

Competition among species is a key force shaping community structure. It has long been recognised that similar coexisting species must present mechanisms that decrease potential competition between them to avoid resource depletion (exploitative competition) or the risk of being injured or killed (interference competition; [1–4]). This fact is particularly evident when the potential for intraguild predation (i.e., an extreme form of interference competition; [5]) is high between species belonging to the same ecological assemblage [5, 6]. Thus, predators can affect individual fitness, as well as population and community processes, through lethal or non-lethal effects, and behavioural or ecological compensation for predation risk should occur [6, 7].

**Fig 1:**
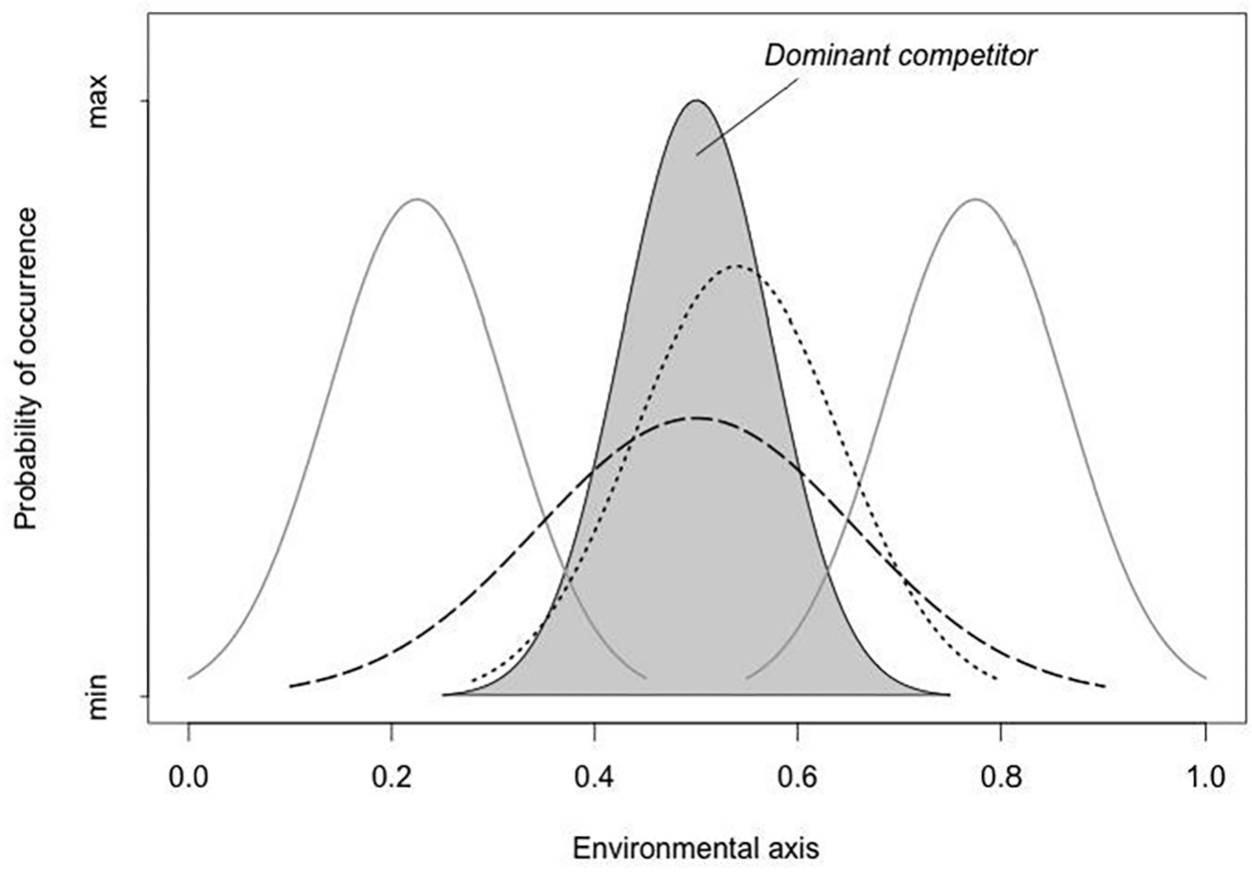
Characteristic of niches for dominant and subordinate species. Graphical representation of characteristics of niches for dominant (shaded area) and subordinate (non-shaded areas) competitors, when interference competition is present. The dominant species would occupy the space that suits its requirements and its niche position and breadth would be determined by the characteristics of the environment (i.e., their fundamental and realized niches would coincide). The subordinate species in the presence of the dominant one, and when interference competition is present, would change its niche to partially or totally avoid competition (encounters in our case) with the dominant species, which would result in wider niche breadth, displaced with regard to its theoretical centre, or even bimodal niches with separate peaks. Thus, when estimating niche parameters over several study areas for the jaguar-puma pair, it is expected that pumas (the subordinate or less competitive species) will present greater variability of parameters (niche centre and/or breadth) than jaguars.

Segregation along the spatial ecological axis of the niche is where more often competition is reflected [8]; also see [9], for evidences of competition on other niche axis). Victims of interference competition might avoid areas of high interference risk by total range segregation, different use of areas or habitats within ranges, or by microhabitat shifting within habitats (e.g., [5, 10–13]). Scale and/or environmental heterogeneity must be taken into account to be able to detect the spatial signature imposed by the biotic interactions within the studied community or pairs of species [14, 15], more even when species are highly similar and interactions are consumer-resource in nature (e.g., [13, 15]). However, in many occasions it is not possible to conduct experiments for logistic or ethical reasons, so disentangling whether segregation in the spatial ecological axis is the result of different habitat preferences or shifts induced by biotic interactions between species frequently remains unanswered. When behavioural or ecological compensation is occurring in subordinate species by virtue of interference competition, theory predicts that their populations are likely to show niche contractions (i.e., specialization) or expansions (i.e., generalization) in the presence of competitors as compared to dominant species (i.e., “realized niches” of dominant species should be closer to “fundamental niches”, while this should not be the case for victim species; [4]). Therefore, examining niche parameters over several different areas would contribute to examine if interference competition explains any spatial segregation pattern found. Thus, as a rule, averaged data of niche parameters from several study areas of the subordinate species would show higher variability than that of the dominant one (Fig 1).

In carnivorous mammals, interference competition (and often its associated intraguild predation) is an extensive phenomenon [16]. In many cases the interaction is asymmetric, with one species usually being the dominant or predator (often the larger one) and the other the subordinate or victim (usually the smaller one; [16, 17]). Several examples demonstrate how the victim species tries to avoid interference competition by segregating along the spatial ecological axis, using different areas or (macro) habitats to successfully avoid or decrease the risk of encounters with the predator species (reviews in [16, 18]). Although it is well established that plant resources, vegetation structure or habitat complexity (i.e., fine-scale spatial approaches) may also moderate the strength of predator–predator interactions under interference competition in arthropod [19] and aquatic systems [10], evidence in terrestrial vertebrate systems is almost non-existent, mainly between similar top predators (see [20–22] for examples in non-closely similar top mammalian predators).

The predator pair of felids, jaguar (*Panthera onca*) and puma (*Puma concolor*), is particularly interesting to address this question in large similar vertebrate potentially competitor top predators. Both species coexist throughout the distribution area of jaguars, and share similar life history traits and behaviours [23]. Both are habitat generalists, found from arid areas to rain forests. Thus, potential for competition between them is high [24], and coexistence has been repeatedly reported in both local community (sensu Hanski & Gilpin [25] definition) and regional metacommunity (sensu Wilson [26]). Under an interference competition scenario, jaguars are dominant over pumas. Despite it is difficult to detect the intraguild predator phenomenon between solitary, forested and low-density species such as jaguars and pumas, there are records of jaguars killing pumas in areas of Brazil, Mexico and Argentina [27–30]. Thus coexisting at a regional scale, theory predicts that pumas should avoid habitats or areas used by jaguars to decrease the risk of encounters with them (i.e., within the local community scale).

Despite these expectations, no study to date has supported the hypothesis of a clear spatial local avoidance of jaguars by pumas [31–35]. In a few cases, usually in which radio-tracking on a limited number of individuals sharing space was carried out, very small differences, almost anecdotal, in (macro-) habitat use were recorded within local community scale studies [36–40]. However, these small differences may well be ascribed to individual variability rather than actual differences between species. The only generally accepted difference found between the two species is that pumas are more tolerant to human-influenced landscapes than jaguars [40, 41]. In fact, in most areas from which jaguars have been extirpated, pumas persist. However, Foster et al. [42] found a contrary result, with jaguars being more tolerant than pumas to human-altered habitats. Thus, the spatial coexistence between these two potentially competing species remains an interesting question to solve. The general result is that they share space (at regional and local scale) without any apparent avoidance. Nevertheless, some authors have proposed that a fine-scale segregation in habitat use (even finer than that of traditional local scale sensu Hanski & Gilpin [25] between the two species should be operating to explain coexistence [31, 40, 42]. As previously stated, results found from other theoretical and empirical studies (see above) suggest that spatial avoidance between species with potential for interference competition may well be scale-dependent, and only observed at the micro-habitat scale.

Here, we first examine whether there is fine-scale habitat segregation between pumas and jaguars, and if so, whether the interference competition risk hypothesis might explain the observed differences.

First, to examine if jaguars and pumas segregate in fine-scale habitat use, we used meta-analysis theory and statistical framework on data collected from a large-scale, non-invasive genetic sampling of faeces within the distribution range of both species, and new, easy-to-use sensor satellite habitat indices available for a great variety of areas and years [43, 44]. We sampled areas with very different environmental conditions to employ traditional comparative study methods. Thus, we carried out a meta-analysis to test for this hypothesis. Meta-analysis is a quantitative method that combines results from different areas or different studies on the same topic to draw a general conclusion and evaluate consistency among study findings [45].

Second, to examine if the interference competition risk hypothesis might explain results, we used the conceptual development of the theory that states that populations that are dominant by virtue of interference competition are likely to show minimal niche contraction or expansion in the presence of competitors as compared to subordinate species ([4]; Fig 1). Thus, if jaguars are the dominant species and pumas the subordinate species, the “realized niche” of jaguars will be closer to the “potential niche” for the spatial axis analyzed in this study, while this should not be the case for pumas. This result would be considered a consequence of the risk for interference competition between the species. Therefore, for data summarized across several study areas, we expected that pumas would present, in general, a greater variability in niche parameters than jaguars in order to avoid sites used by jaguars (Fig 1). To test these predictions, we used a multivariate method, the Outlying Mean Index (OMI; [46]), to estimate niche parameters for both species in several study areas. OMI analyses address the question of niche breadth and niche separation by measuring the distance between the mean habitat conditions used by the species (species centroid), and the mean habitat conditions of the sampling area (origin of the niche hyperspace).

## Materials and Methods

### Species sampling

Pumas and jaguars were systematically sampled by slowly walking on dirt roads and animal- and human-made trails searching for faeces of the two species in 12 different areas (five Mexico: all in Yucatan peninsula, and seven in Brazil: four Amazon, one in Pantanal, one in Cerrado and another in Caatinga; S1 Study Areas and Methods; S1 Fig). Faeces surveys followed by genetic analyses are a very convenient method for sampling carnivores in fine-scale habitat studies because they provide an accurate way of detecting elusive species in large scale surveys [47]. On a few occasions, faeces were opportunistically collected during other research activities or moving through the study areas. Except on rare occasions, faeces were collected during the dry season. Each area was sampled from one to five times between 2004 and 2012. Caiman, Capivara and Caobas were sampled in one year, Calakmul, Petcacab, Maraca and Uatumã in two years, Eden, Zapotal, Virua and Emas in three years, and Ducke in five years. Since we always simultaneously sampled both species in each area and year, we assumed that this fact did not influence our results. In Refúgio Caiman, Serra Capivara, and Emas National Park faeces were collected with the help of scat detector dogs [48].

The location of faeces was georeferenced with the aid of GPS (® Garmin), and an accuracy of 3–5 m. For fresh samples, a small portion was initially stored in 96% ethanol for 24–48 hours and then transferred to silica gel for storage. Dried samples were directly stored in silica until genetic analyses were conducted.

Sampling in Brazil were carried out under licenses no. 11214 and no. 13781 of ICMBio, and nº 131/2005 CGFAU/LIC, 13883–1 SISBIO and 15664–1 SISBIO of the Instituto Brasileiro do Meio Ambiente–IBAMA, and at the Mexican sites under the licence SGPA/DGVS/549 of the Dirección General de Vida Silvestre (Semarnat). Owner, mayors or directors of private communal lands (Ejido Caoba and Ejido Petcacab in Mexico) or reserves (El Edén and Zapotal in Mexico, Refúgio Ecológico Caiman in Brazil) provided with permission for sampling as well. Faecal samples were exported from Brazil to Spain for genetic analysis under IBAMA/CGEN Autorização de Acesso licence nº 063/05 and IBAMA/CITES export licences nº 0123242BR, 08BR002056/DF and 09BR003006/DF, and from Mexico to Spain under the export licences nº MX33790 and MX42916 of the Secertaria de Medio Ambiente/CITES. Since we conducted a non-invasive sampling of faeces, the study did not need any approval by an Institutional Animal Care and Use Committee or equivalent animal ethics committee.

### Genetic analyses of faeces

DNA was extracted from faecal samples using protocols based on the GuSCN⁄silica method [49] and further purified and concentrated through ultrafiltration using Microcon-30 (Millipore). Species identification was performed using previously developed species-specific primers [50]. All molecular analyses were undertaken in the Molecular Ecology Laboratory of the Doñana Biological Station (LEM-EBD).

### Fine-scale habitat characteristics of areas used by jaguars and pumas

One limitation in the analysis of habitat use and selection for species living in remote areas is the lack of data for a detailed characterisation of their habitats. Although global land-cover products can provide information on the distribution of broad vegetation categories (e.g., forests vs. agricultural areas), this information is too coarse to detect heterogeneity in environmental characteristics, including fine-scale variations in the vegetation composition and structure (reviewed in [44]). On the other hand, performing vegetation classifications *ad-hoc* is often unfeasible in habitat studies involving large spatial areas or many different study areas. To overcome these problems, we designed an alternative approach focusing on differences in synoptic indicators of the functional characteristics of ecosystems between sites selected by jaguars and pumas as a surrogate of habitat attributes. Therefore, we characterised the sites used by both species based on spectral indices of ecosystem functioning calculated from remote sensing [51]. These indices are integrative descriptors of the land surface affected by both structural and functional characteristics of the vegetation, including the vegetation cover, plant canopy architecture, species composition, phenology and productivity (see [52], for further details). Furthermore, they provide consistent information with global coverage at fine spatial scales, making them highly useful for analysing remote, broad and separated regions using a uniform methodology.

We specifically used time series of the Enhanced Vegetation Index (EVI; [53]) to characterise the functional properties of ecosystems (seven different functional attributes were calculated; see S1 Study Areas and Methods for a detailed description of the procedure) around each site where either of the two species were located. The seven different descriptors aimed to capture the potential differences between sites in the seasonal vegetation dynamics and the inter-annual variability and were selected attending to their capacity to synthesize the properties of the EVI time series. The annual EVI integral (EVIi), a surrogate of the annual ecosystem primary productivity, was estimated as the mean of all EVI values. The inter-annual variability in productivity (cvEVIi) was estimated as the coefficient of variation of the annual EVI integral. The average maximum EVI (EVImax) and the average minimum EVI (EVImin) were estimated as measures of the annual primary productivity peaks and decreases, respectively. The average relative difference between the annual maximum and minimum (EVIrel), and the average coefficient of variation between values within the year (cvEVIi) were both calculated as surrogates for vegetation seasonality. Finally, the circular dispersion in the date of maximum EVI was estimated as a measure of inter-annual fluctuations in the period of maximum vegetation productivity (rEVIvec).

### Meta-analysis

We used so-called meta-analytic thinking [54] to examine if jaguars and pumas differed in fine-scale habitat use (i.e., primary productivity in 250-m cells; see above). Meta-analysis allows for the comparison of data through effect size estimates [55]. We used the Hedges’ d to calculate the effect size, including a correction when sample size was below 20 [45, 55], which was the case in some of our study areas (S1 Table). Hedges’ d expresses in standard deviations the magnitude of response of comparisons between two groups. Because we did not expect that jaguars and pumas use sections of higher or lower primary productivity in the same way in all study areas, which were located in very different biomes, we calculated the absolute difference in the EVI index means rather than the difference in any one direction (e.g., [56]). Our hypothesis was simply that jaguars and pumas differ in fine-scale habitat use when inhabiting the same study areas, making no inference about the ultimate environmental causes of such differences. Therefore, absolute values of effect sizes were an appropriate measure for testing our hypothesis.

For comparison between species, we ran separate random-effects meta-analyses for each focal EVI variable using a restricted maximum-likelihood estimation with the Metafor R package [57, 58]. Random-effects meta-analysis uses the more reasonable assumption that each study has a ‘true’ effect size different from each other [58, 59]. When more than one annual survey was carried out in a given study area, samples were pooled for analysis. Study areas with less than two sampling cells for any species were not considered for analyses, and we only considered each cell once for each species (note that sometimes there were >1 faeces per cell).

Additionally, we calculated a composite effect size [60] to examine the magnitude of the overall response in fine-scale habitat use between species. For this purpose, we used a flexible meta-analytical approach [59, 61], considering the general effect sizes for each study area and species. This approach allows the use of multiple non-independent effect sizes (i.e., different EVI indices in our case) obtained for the same study areas allowing an increase in the statistical power of the comparison and a unique estimate of effect sizes for each study area. We used a Markov chain Monte Carlo algorithm to fit generalized linear mixed-effects models and estimate parameters (package MCMCglmm in R version 3.1.0; [62, 63]).

### OMI analyses

Niche parameters were estimated by the Outlying Mean Index (OMI; [46]). In contrast to other multivariate methods, OMI describes the species response whether it is linear or unimodal, giving equal weight to species-rich and species-poor sites. Its interpretations are robust to multicollinearity among the explanatory variables [46]. OMI measures the marginality (so-called OMI) of species, or the distance between the average environmental conditions (in our case) used by a species and the mean environmental conditions of the sampling units of the study area. A high marginality indicates that a species is found under atypical environmental conditions within the study area, whereas a low marginality indicates that there is no difference between the overall environmental conditions and those where the species is found. The OMI analysis also provides an index of tolerance as a measure of niche breath. High tolerance values indicate that the species is distributed along a variety of environmental conditions, while low values imply that the species is distributed along a more limited range of environmental conditions. A third niche parameter is the residual tolerance. The residual tolerance index indicates the variance in species niche not taken into account by the marginality axis. Thus, this index helps to determine the influence of the tested environmental conditions on the distribution of the species. Low values indicate that the relationship between the study's environmental conditions and the species distribution is high, whereas high values indicate that they are weakly related. Additionally, the OMI analysis also provides an inertia estimate, and its value represents the total variance of the environmental table weighted by the species distribution profile. This variability is decomposed into OMI+tolerance+residual tolerance (expressed as percentages of inertia), so the individual proportion of this variability associated with the three indices may be directly related with the specific parameter of interest examined in this study (marginality or tolerance).

OMI uses an environmental matrix with the values for all the sampling sites in the study area for the selected environmental variables (EVI indices in our case), and a species matrix that included the number of samples of each species per each sampling site (number of faeces in our case). Sampling sites were those 250-m grid cells that were intersecting transects. On average, for the 12 study areas we obtained information on environmental values (i.e., EVI indices) for 570±444.7 grid cells (range = 127–1561).

OMI analyses were conducted using the ADE-4 package [64] of the R Statistical Package [57], and differences between the two species in niche parameters were examined by t-tests, once data were log- or arcsin-transformed to comply with normality assumptions.

To graphically represent the magnitude of the differences when comparing jaguars and pumas for OMI results (marginality, tolerance and residual tolerance), we employed a commonly used metric in ecology, the log response ratio (*lr*; [55]):

$$lr=\text{ln}(\frac{X^{j}}{X^{p}})$$

where X^j^ and X^p^ are the mean values for jaguars and pumas, respectively. Thus, in our case, *lr* would indicate how many times a given niche parameter of jaguars (positive values) or pumas (negative values) would be higher than that of the other species. General trends in variability of data (OMI and tolerance) for each species independently were also represented by constructing standard boxplots.

## Results

### Sample sizes

For the 12 study areas, the mean number of faeces was 30.1 (range = 2–67) and 28.9 (range = 8–64) for jaguars and pumas, respectively (S1 Table). Once we removed duplicate samples for the same cells, the mean number of sampled cells was 27.4 (range = 2–61) and 27.3 (range = 8–60) for each species, respectively.

### Fine-scale habitat segregation

Effect sizes on productivity indices of the areas considered as being used by pumas and jaguars for the 12 study areas were only significantly different from zero in two out of 12 areas for meanEVIi, in one area for meanEVIcv, two areas for meanRVIrrel, two areas for rEVIvec, no area for cvEVIi, three areas for meanEVImin, and one area for meanEVImax (S2 Fig). However, meta-analysis detected significant differences for the seven EVI indices considered, and effect sizes ranged between 0.249 and 0.382 (Table 1). There was no significant heterogeneity among study areas in effect sizes (Table 1), and therefore it was not necessary to look for a moderator variable that could explain observed differences among the areas. Considering all the variables together, meta-analysis found that jaguars and pumas segregated by 0.318 (95% CI = 0.197–0.457) standard deviations in fine-scale habitat use.

**Table 1:** Effect sizes for EVI indices.

Meta-analysis ID	Effect size (Hedges’ d)	95% CI	Heterogeneity			
			tau	Q	df	p
*MeanEVIi*	**0.256**	**0.074–0.437**	0.072	12.74	11	0.3106
*MeanEVIcv*	**0.318**	**0.144–0.493**	0	5.76	11	0.8886
*MeanEVIrrel*	**0.382**	**0.179–0.586**	0.158	13.18	11	0.2820
*rEVIvec*	**0.249**	**0.079–0.419**	0	5.81	11	0.8857
*cvEVIi*	**0.260**	**0.086–0.434**	0	2.53	11	0.9956
*MeanEVImin*	**0.263**	**0.090–0.436**	0	12.33	11	0.3390
*MeanEVImax*	**0.342**	**0.141–0.543**	0	13.23	11	0.2784

Effect size estimates for the effect of species (jaguar and puma) on several EVI index values for 12 areas where jaguar and puma faeces were collected. The number of effect sizes (k) used for meta-analyses was 12 for all variables. Heterogeneity (tau) of the tests is indicated in the table. Statistically significant effect sizes are in **bold**. The Hedges’ d indicates the number of standard deviations that separate the species in the value of the variable considered. A value of zero in effect size indicates no difference in the value of the variable between species.

### Niche variability

**Fig 2:**
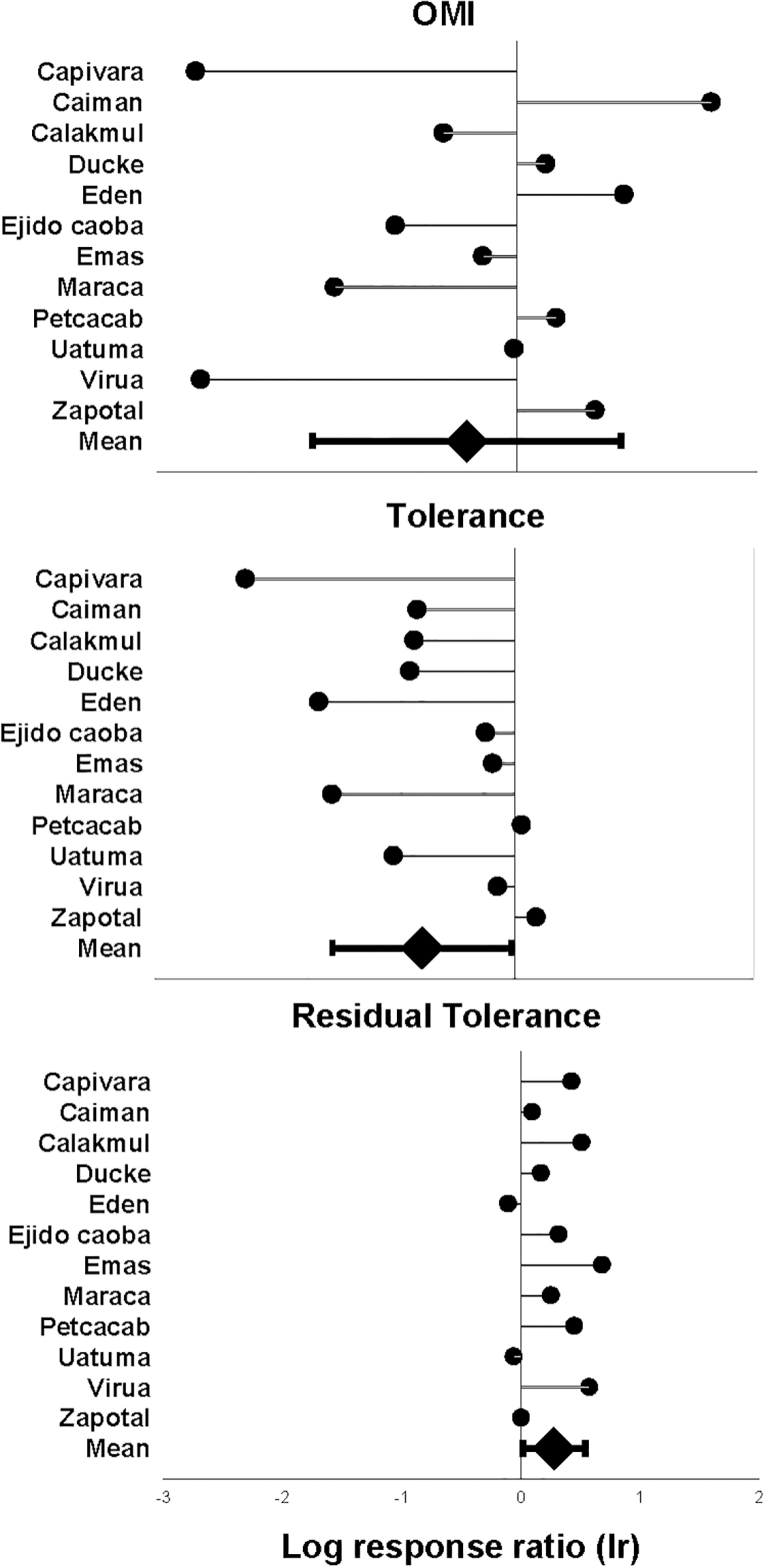
Log response ratio for niche parameters. Log response ratio (*lr*) for OMI (marginality), tolerance and residual tolerance for showing differences in these indices between jaguars and pumas for the 12 study areas. On the graph, zero values of *lr* means that the two species had identical index values, and a value of 1 (absolute value) means that jaguars (positive values) or pumas (negative values) would have an index value 2.7 times higher than the other species for a given index and study area. Mean *lr* (±SD) is shown at the bottom of each graph.

**Fig 3:**
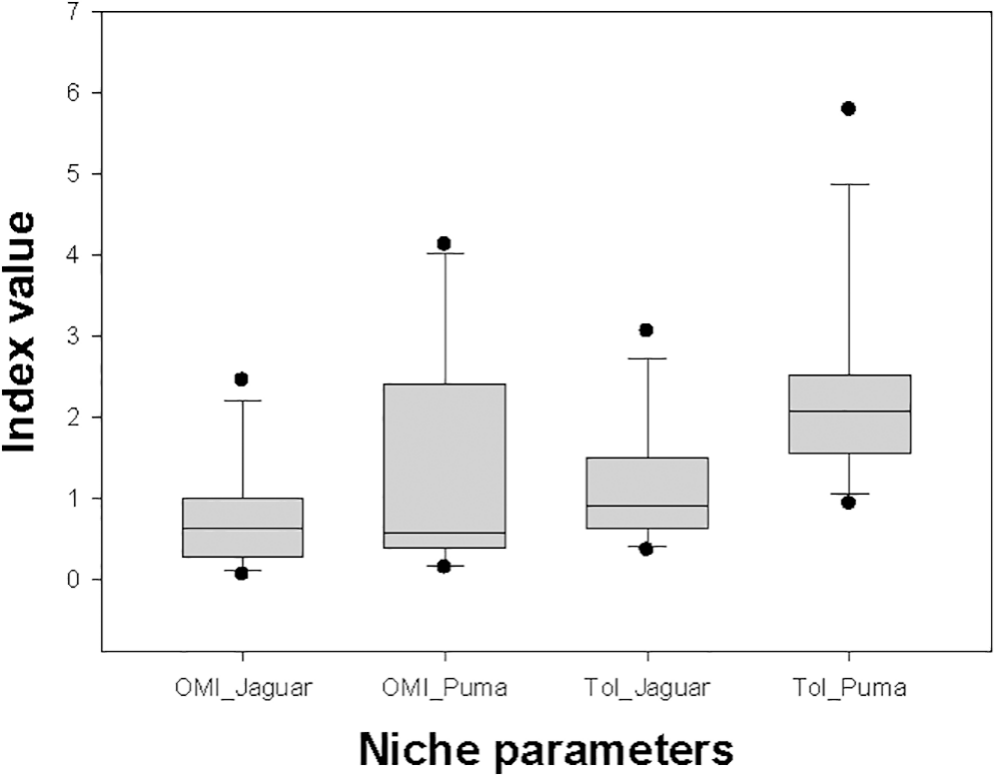
Data dispersion of niche parameters. Boxplots show differences in dispersion of overall data for relevant niche parameters (OMI or marginality, and tolerance, Tol) of jaguars and pumas for the 12 study areas. The box indicates the 25th and 75th percentiles, a line within the box marks the median; error bars indicate the 90th and 10th percentiles, and points values outside the last percentiles. Despite similar OMI median values, variability of puma data is higher than that of jaguars, as predicted under the interference competition hypothesis, with pumas being the subordinate species in the interaction

On average, marginality for pumas was 1.51 times higher than that for jaguars, but there was no consistent pattern across areas. Marginality was higher for pumas in seven areas and for jaguars in five areas (Fig 2), resulting in non-significant overall differences (Table 2). However, although median values were quite similar between species (slightly lower for pumas), dispersion of the marginality data was much higher for pumas than for jaguars (Fig 3). Therefore, the central position of the realized niches of pumas was more variable than that of jaguars.

**Table 2:** Mean values of niche parameters for jaguars and pumas.

	Inertia		OMI		Tol		RTol		*OMI*		*Tol*		*RTol*	
	Mean	SD	Mean	SD	Mean	SD	Mean	SD	Mean	SD	Mean	SD	Mean	SD
Jaguar	6.76	2.11	0.77	0.68	1.11	0.77	4.88	1.60	11.68	10.16	15.71	8.35	72.51	12.28
Puma	7.20	1.78	1.36	1.42	2.24	1.25	3.59	0.76	17.14	16.35	30.82	12.05	52.04	13.69
	0.66, 0.516		0.93, 0.363		**3.53, 0.002**		**2.367, 0.027**		0.997, 0.329		**3.463, 0.002**		**3.929, 0.001**	

Mean values of niche parameters for jaguars and pumas in the 12 study areas sampled. *OMI*, *Tol* and *RTol* in italics represent the corresponding percentages of variability (i.e., of the inertia value) attributed to the corresponding niche parameter. The values of the t statistic, and P-value are given in the last row for comparison between species (degree of freedom were always 22, and the statistically significant differences are indicated in **bold** if P<0.05). OMI = outlying mean index or marginality; Tol = tolerance index, RTol = residual tolerance index.

Tolerance was consistently higher in most study areas (10 out of 12) for pumas, and overall differences were statistically significant and 2.2 times higher for pumas than for jaguars (Fig 2; Table 2). The boxplot representations of the data confirmed the trends for both species (Fig 3). Thus, the realized niches of pumas were wider and more variable than those of jaguars.

Residual tolerance (i.e., the variance in the species niche that is not taken into account by the marginality axis) was higher in jaguars for most study areas (9 out of 12), being on average 1.3 times higher than for pumas (Fig 2), and the difference was statistically significant (Table 2).

Furthermore, on average inertia was quite similar in both species; however, 73% of this variability was from residual tolerance for jaguars, which was significantly higher than that for pumas (52%). Another 31% of variability of puma data came from tolerance (i.e., variability in niche breadth), which was significantly higher than for jaguars (16%; Table 2). There were no significant differences in percentage of inertia coming from marginality (Table 2). Again, the index related to variability in niche breadth was higher for pumas than for jaguars.

## Discussion

Segregation in the spatial niche axis is a well-known mechanism of decreasing competition among ecologically-similar species [5, 10, 11, 13]. A given species uses different areas or habitats to avoid prey depletion by the dominant exploitative species or more successfully refusing risk of being injured or killed by the dominant interference species [6, 7, 11]. In any case, the first step is to detect if there is spatial segregation between a pair of potentially competing species. The meta-analysis performed in this study on data collected in 12 study areas showed that two coexisting top predators, jaguars and pumas, used areas with different microhabitat characteristics as measured by the EVI indices. This result confirmed our first prediction, which stated that the two species would segregate in fine-scale space use. However, the relevance of this result goes further, as in our knowledge, this is the first study showing a fine-scale habitat segregation between two top and ecologically similar vertebrate predators, and besides, the spatial segregation was found in much finer scale than usual (i.e., local community scale sensu Hanski & Gilpin [25]).

Some studies have shown that competing vertebrate predators may use different types of macrohabitats (review in [16, 18]), but only a couple of them have shown the importance of environmental heterogeneity to allow for the coexistence of subordinate and dominant predators at really small spatial scales. Swift fox (*Vulpes velox*) survival was higher in areas of lower shrub density despite higher density of the dominant interference predator, coyote (*Canis latrans*) [20]. In scrubland areas where Iberian lynx (*Felis pardina*) and the Egyptian mongoose (*Herpestes ichneumon*) coexist, the latter, the inferior interference predator, uses denser parts of the scrubland than in areas where the former is not present [21]. These examples and the results of this study suggest that the subordinate predator species can avoid the dominant species by fine-shifting micro-habitat use. This behaviour has been previously shown for prey species [7], but not for intermediate predators [11]. Differences may be very subtle and barely measurable under the traditional definitions of (macro) habitat types.

In addition to the ecological results, we also showed that remote sensing may help with these issues (also see [65]). The use of ecosystem functioning variables provided by remote sensing has allowed the detection of subtle, but clear, differences in the environmental characteristics of the habitats used by two similar competing top predators, which, as a rule, until now had been thought to use the same habitats in areas of coexistence (e.g., [31–36]). Thus, we can also conclude that detecting spatial segregation between jaguars and pumas was scale-dependent, and as stated, this fact had not been previously reported in other species of coexisting ecologically similar top predators. The theoretical work of Araújo & Rozenfeld [15] and the empirical study of Swanson et al. [22] with carnivores in Africa show that interactions of the type dominant-subordinate can be manifest in small scale and not in larger ones.

Some other considerations should be made regarding the spatial resolution we selected for the fine-scale habitat use analyses, in order to be confident that results were biologically meaningful for the question posed. We assigned faeces to grid-cells with sides of approximately 250 m (i.e., 6.25 ha), where EVI indices where calculated. This resolution was considered adequate because smaller areas could have shown differences for the particular points selected for faecal deposition [66].

Results from OMI analyses of the spatial ecological niche of both top predators further supported the interference competition risk hypothesis to explain the found segregation pattern. Under an interference competition scenario, theory predicts that the dominant species should present more similar realized and fundamental niches when studied in several study areas, whereas the subordinate species should present wider and more variable observed niches (Figs 1 and 3). The results for both species in the 12 study areas confirmed these predictions. As predicted, pumas had more highly variable observed niche parameters (marginality and tolerance) than jaguars, which were considered an indirect measure of interference competition with a dominant species. This result is also in agreement with the limiting similarity hypothesis [2], which states that species can be no more similar in their utilizations than a certain degree, if they are to coexist. A further test would be to study spatial niche parameters of pumas in areas where jaguars are absent. In these situations, pumas should present less variable niche parameters, and their realized niches should be closer to the fundamental niches.

Exploitation competition hypothesis might also explain the observed patterns about habitat segregation between jaguars and pumas (i.e., differences in habitat segregation might be due to differences in prey availability for each species). To quantify accurately prey availability is a very difficult task, and far from being feasible for the species studied here and the large scale of this study. However, a number of facts indicate that prey abundance and composition might be similar in cells used by the two predators. In a recent review, Martínez-Gutiérrez et al. [67] identified the most frequently consumed prey by jaguars and pumas in the Neotropics, and most prey (57%, n = 14) were common to both felids. All the most consumed prey of both species have home ranges >6 ha (i.e., equal or higher areas than our grid-cells), and occur in the same general macrohabitat types shared with the predators (e.g., [68–70]). Furthermore, it has been previously and repeatedly shown that, when coexisting, the two species use the same areas and macrohabitats (see references above). This was also the case in our 12 study areas, where the mean distance between jaguar and puma locations averaged 1306 m (SD = 813, range = 264 and 2902 m; authors unpubl. data), while home range sizes for both species are usually larger than 30–50 km^2^ (e.g., [31, 39, 71, 72]). Therefore, we can assume that sites used by jaguars and pumas generally have the same prey abundance and composition, and differences in prey availability should not explain results on the fine-scale habitat use observed in this study.

Interference in carnivorous mammals is favoured when its cost is small, its effect is high, and the resource overlap with the species interfered against is high [17], although it can be strong even in cases with minimal diet overlap [73]. Potential food resource overlap between jaguars and pumas is always high [67]. Therefore, interference competition by jaguars is likely to be a strategy alternative to higher exploitation efficiency by pumas. However, there are some situations in which the cost of interference competition may be high for jaguars. Both species greatly vary in body mass between areas, with differences between them being minimal in some parts of the coincident distribution area, where male pumas may even be larger than female jaguars (e.g., Central America; [39, 74]). Thus, the strength of the interaction between jaguars and pumas may be mediated by the body mass differences between them and this may explain in part (but see below) differences found between our study areas. Unfortunately, there is no specific information on body mass of male and female jaguars and pumas for all the study areas and we could not examine this issue.

Along this same line, interference competition may also be manifest with different strength along resource and habitat gradients, and the interference competitor may exclude the exploitation competitor at the rich end of the resource gradient, whereas the reverse would be true at the poor end of the gradient ([4]; also see [21, 22, 75], for examples with the pairs Iberian lynx-Egyptian mongoose and African lion (*Panthera leo*)-cheetah (*Acynonix jubatus*)). For example, in areas rich in food resources jaguars may attain high density and thus have a greater impact on puma space use and abundance. In this case we predicted 1) a measurable negative effect of jaguars on puma abundance, and 2) a spatial segregation in the local community scale. However, if food resources are poor, we predicted that jaguars would be in low abundance and pumas could attain higher abundances using most of the area, and a fine-scale habitat segregation between both species would not exist. Thus, results found for particular areas might be explained by differences in abundances of the two species.

## Supporting Information

S1 Fig Location of the areas sampled. Areas of Latino America where faecal samples were collected for studying microhabitat segregation between jaguar and pumas between 2004 and 2012. 1 = Zapotal, 2 = El Eden, 3 = Ejido Petcacab, 4 = Ejido Caoba, 5 = Calakmul, 6 = Maracá Ecological Station, 7 = Viruá National Park, 8 = Uatumã Biological Reserve, 9 = Ducke Reserve, 10 = Serra da Capivara National Park, 11 = Emas National Park, 12 = Refúgio Ecológico Caiman.
(PDF)

S2 Fig Hedges’ d effect sizes. Absolute values of Hedges’ d effect sizes and their 95% confidence intervals of the comparison between values of several EVI indices in 250 m cells used by jaguars and pumas for each study area included in this study.
(PDF)

S1 Study Areas and Methods Supporting information about the Study area and EVI indices. Description of the 12 study areas and characterisation of the functional properties of ecosystems measured with the Enhanced Vegetation Index around each site where jaguars and pumas were located.
(PDF)

S1 Table Mean values of EVI indices. Jaguars and puma mean±SE values for several EVI indices for 250 m cells in the different study areas included in the study.
(PDF)
