# Supplementary material for: Fine-Scale Habitat Segregation between Two Ecologically Similar Top Predators
Source: PLoS One. 2016 May 17;11(5):e0155626. doi: 10.1371/journal.pone.0155626 (PMC4871328; doi:10.1371/journal.pone.0155626)

S1 Fig. Location of the study sites. Faecal samples were collected for studying microhabitat segregation between jaguar and pumas between 2004 and 2012 at twelve study sites. 1= Zapotal; 2= El Eden; 3= Ejido Petcacab; 4= Ejido Caoba; 5= Calakmul, 6= Maracá Ecological Station; 7= Viruá National Park; 8= Uatumã Biological Reserve; 9= Ducke Reserve; 10= Serra da Capivara National Park; 11= Emas National Park; 12= Refúgio Ecológico Caiman. Land Cover types are a reclassification of GlobCover 2009 V.2.3. Wource GlobCover product copyrighted by ESA 2010 and UCLovain.

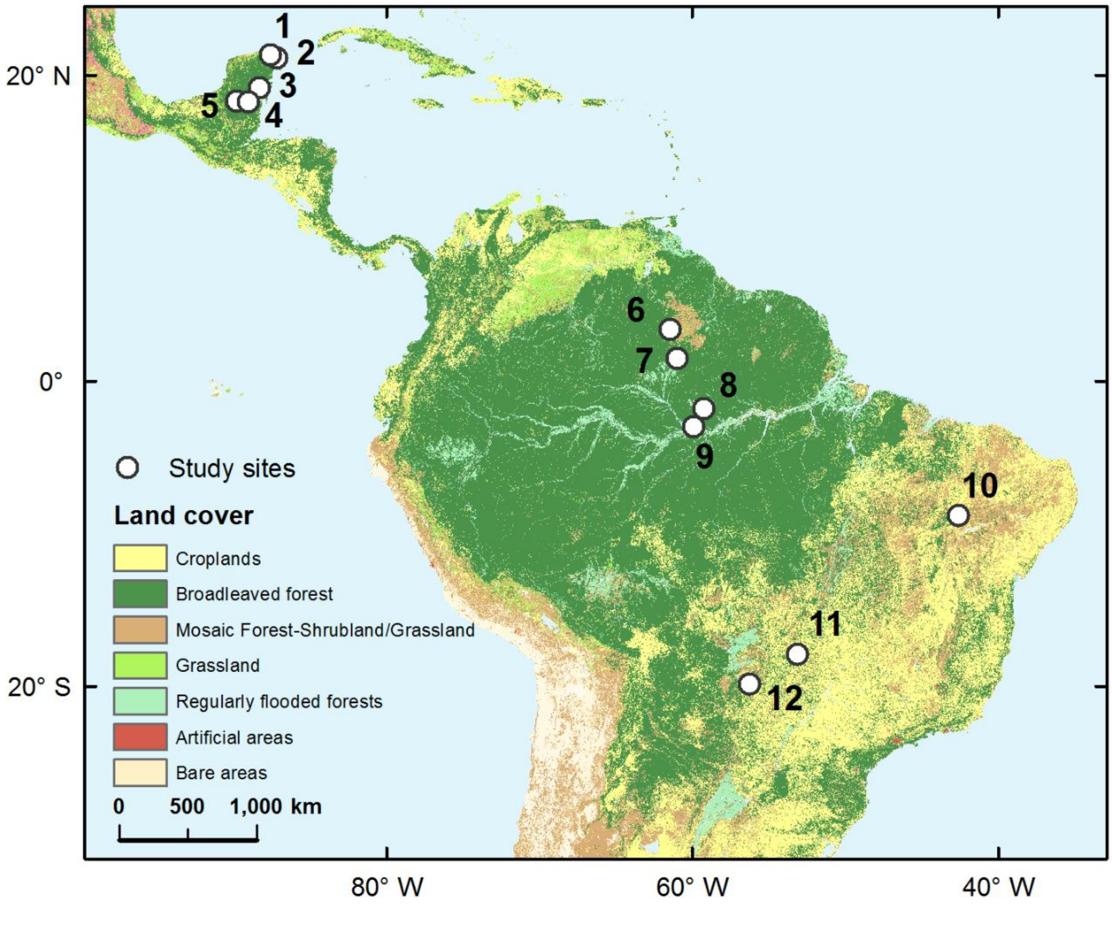

Supplement: S1 Fig — Areas of Latino America where faecal samples were collected for studying microhabitat segregation between jaguar and pumas between 2004 and 2012. 1 = Zapotal, 2 = El Eden, 3 = Ejido Petcacab, 4 = Ejido Caoba, 5 = Calakmul, 6 = Maracá Ecological Station, 7 = Viruá National Park, 8 = Uatumã Biological Reserve, 9 = Ducke Reserve, 10 = Serra da Capivara National Park, 11 = Emas National Park, 12 = Refúgio Ecológico Caiman. (PDF) [file pone.0155626.s001.pdf]
