# Supplementary material for: Fine-Scale Habitat Segregation between Two Ecologically Similar Top Predators
Source: PLoS One. 2016 May 17;11(5):e0155626. doi: 10.1371/journal.pone.0155626 (PMC4871328; doi:10.1371/journal.pone.0155626)
Supplement: S2 Fig — Absolute values of Hedges’ d effect sizes and their 95% confidence intervals of the comparison between values of several EVI indices in 250 m cells used by jaguars and pumas for each study area included in this study. (PDF) [file pone.0155626.s002.pdf]

S2 Fig. Hedges' d effect sizes. Absolute values of Hedges' d effect sizes and their 95% confidence intervals of the comparison between values of several EVI indices in 250 m cells used by jaguars and pumas for each study area included in this study.

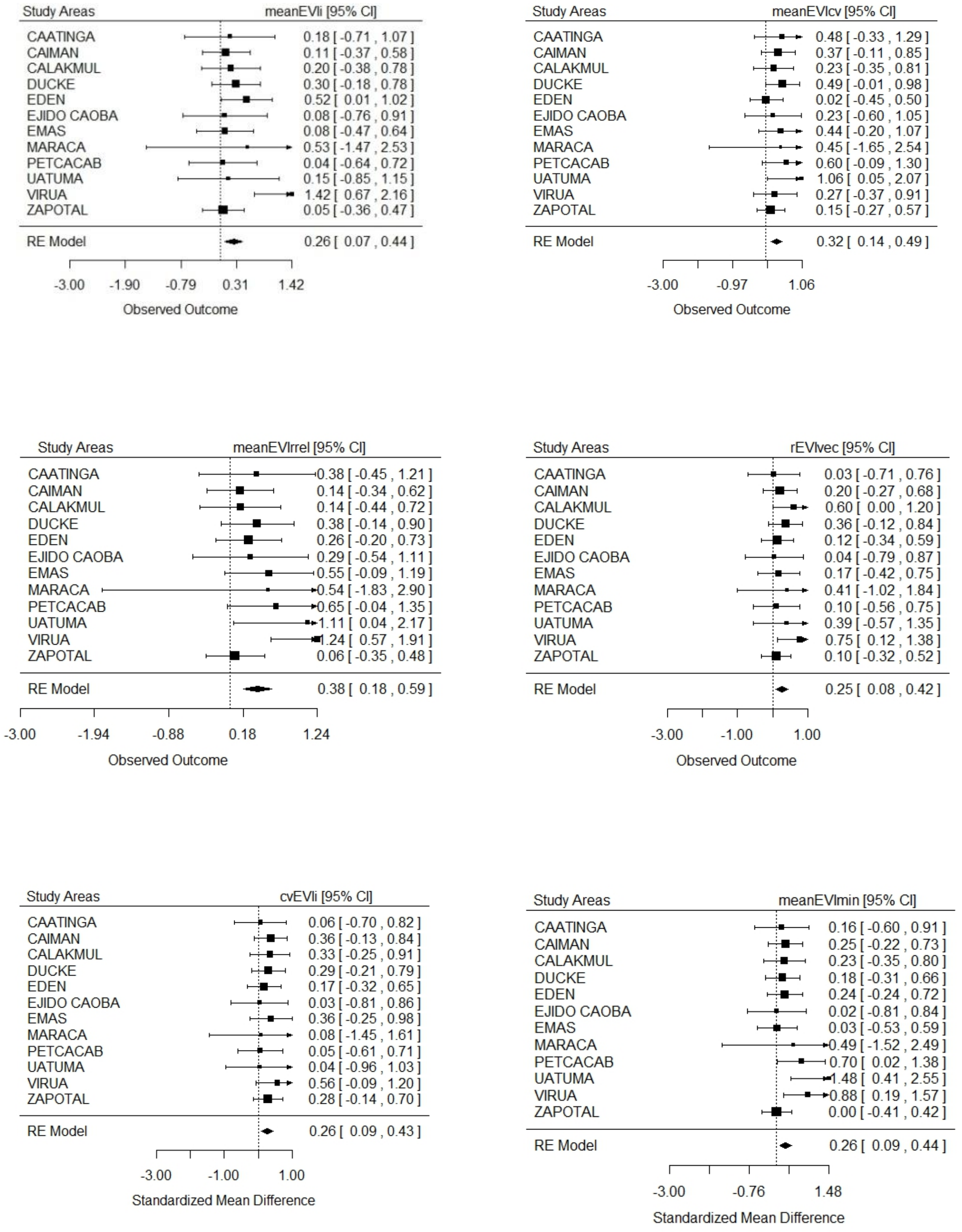

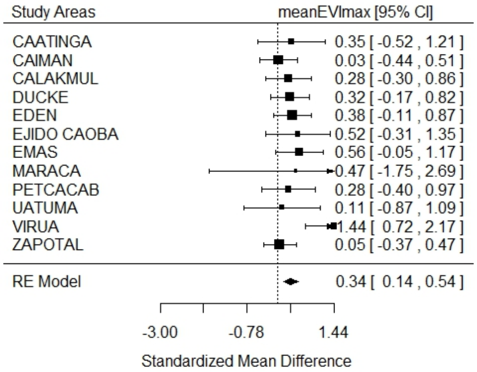
Study Areas
meanEVImax [95% CI]
CAATINGA
0.35 [-0.52 , 1.21]
CAIMAN
0.03 [-0.44 , 0.51]
CALAKMUL
0.28 [-0.30 , 0.86]
DUCKE
0.32 [-0.17 , 0.82]
EDEN
0.38 [-0.11 , 0.87]
EJIDO CAOBA
0.52 [-0.31 , 1.35]
EMAS
0.56 [-0.05 , 1.17]
MARACA
0.47 [-1.75 , 2.69]
PETCACAB
0.28 [-0.40 , 0.97]
UATUMA
0.11 [-0.87 , 1.09]
VIRUA
1.44 [0.72 , 2.17]
ZAPOTAL
0.05 [-0.37 , 0.47]
RE Model
0.34 [0.14 , 0.54]
-3.00
-0.78
1.44
Standardized Mean Difference
