# Supplementary material for: Fine-Scale Habitat Segregation between Two Ecologically Similar Top Predators
Source: PLoS One. 2016 May 17;11(5):e0155626. doi: 10.1371/journal.pone.0155626 (PMC4871328; doi:10.1371/journal.pone.0155626)
Supplement: S1 Study Areas and Methods — Description of the 12 study areas and characterisation of the functional properties of ecosystems measured with the Enhanced Vegetation Index around each site where jaguars and pumas were located. (PDF) [file pone.0155626.s003.pdf]

S1 Study area and methods. Supporting information about the Study area and EVI indices. Description of the 12 study areas and characterisation of the functional properties of ecosystems measured with the Enhanced Vegetation Index around each site where jaguars and pumas were located.

Study areas

Faecal samples were collected in five areas from the Yucatan Peninsula, Mexico (Calakmul, 18º 11’ 05” N, 89º 44’ 49” W; El Eden, 21º 13’ N, 87º 11’ W; Zapotal, 21º 20’ 25” N, 87º 36’ 20” W; Ejido Caoba, 18º 14’ N, 89º 03’ W; and Ejido Petcacab, 19º 17’ 15” N, 88º 13’ 33” W), four in the Amazon, Brazil (Ducke Reserve, 02º 55’ S, 59º 59’ W; Uatumã Biological Reserve, 1º 46’ S, -59º 16’ W; Viruá National Park, 1º 29’ 9” N, 61º 2’10” W; and Maracá Ecological Station, 3º 24’ 26” N, 61º 29’ 13” W), one in the Pantanal, Brazil (Refúgio Ecológico Caiman, 19° 57’ S, 56° 18’ W), one in the Cerrado, Brazil (Emas National Park, 18º 19’ S, 52º 45’ W), and one in the Caatinga, Brazil (Serra da Capivara National Park, 8º 26’ S, 42º 19’ W). The nearest areas sampled were El Edén and Zapotal situated at 40 km, and the furthest ones the Zapotal and Serra da Capivara National Park situated at more than 5.900 km (S1 Fig).

The Yucatan peninsula is characterised by tropical rain forest and semi-evergreen forest, and, to a lesser extent, tropical deciduous forest and seasonally flooded forest. The Ejido Caoba and Ejido Petcacab are characterised by mixed tropical rain forest and semi-evergreen forest. Calakmul is a semi-evergreen and seasonally flooded forest, whereas Zapotal and El Edén present a mix of semi-deciduous tropical forest and secondary vegetation, seasonally flooded forest, savannas and aquatic vegetation.

In the Amazon Basin, Ducke and Uatumã are covered by wet tropical rainforest, while Viruá and Maracá are characterised by mosaics of vegetation formed by

transitions between savannas and tropical upland forest. The Refúgio Ecológico Caiman is a cattle ranch and ecotourism business located in the Pantanal wetlands with a mosaic of floodplains, grasslands, savannas, riparian forests and exotic pastures. Emas National Park is situated in the Cerrado grasslands, where large tracts of grassland plains are interspersed with patches of shrub fields, marshes, and riparian forest. It is virtually surrounded by crop plantations. The Serra da Capivara National Park is situated in the semi-arid Caatinga biome and is predominantly covered by 6-10 m tall shrubby vegetation.

Fine-scale habitat characteristics of areas used by jaguars and pumas

We used time series of the Enhanced Vegetation Index (EVI; [1]) to characterise the functional properties of ecosystems around each site where jaguars and pumas were located, according to the following procedure. First, the complete time series of EVI values was extracted for each species location for the period between 2001 and 2012. This was achieved using EVI data provided by the Moderate-Resolution Imaging Spectroradiometer (MODIS) on-board the Terra satellite from NASA. The information was extracted at the MODIS nominal resolution of 250 m to characterise the area around each jaguar and puma location. The influence of atmospheric signal effects and view-angle effects was minimized using the maximum value of the 16-day constrained-view angle composite product, resulting in a total of 23 EVI values per pixel per year. These values were explored and filtered using the “pixel reliability summary quality” layer to eliminate data potentially affected by clouds [2]. Missing values between dates with reliable data were then filled with the midpoint EVI value between the two dates. Finally, years that still contained >15% missing data were not included in subsequent analyses. The time-series analysis was needed to characterize intra- and inter-annual vegetation dynamics. It is true that dramatic changes in vegetation such as clear-cuts

would affect interannual indices. However, we made sure that no significant changes in vegetation structure have occurred in any of these areas during the study period.

References

1. Huete A, Didan K, Miura T, Rodriguez EP, Gao X, Ferreira LG. 2002. Overview of the radiometric and biophysical performance of the MODIS vegetation indices. *Remote Sensing of Environment* 83, 195–213. Retrieved from <Go to ISI>://000179160200014.

2. Solano R, Didan K, Jacobson A, Huete A. 2010. *MODIS Vegetation Index User's Guide* (Vol. 2010). Retrieved from http://vip.arizona.edu/documents/MODIS/MODIS_VI_UsersGuide_01_2012.pdf
