# Supplementary material for: Fine-Scale Habitat Segregation between Two Ecologically Similar Top Predators
Source: PLoS One. 2016 May 17;11(5):e0155626. doi: 10.1371/journal.pone.0155626 (PMC4871328; doi:10.1371/journal.pone.0155626)
Supplement: S1 Table — Jaguars and puma mean±SE values for several EVI indices for 250 m cells in the different study areas included in the study. (PDF) [file pone.0155626.s004.pdf]

S1 Table. Mean values of EVI indices. Jaguars and puma mean±ES values for several EVI indices for 250 m cells in the different study areas included in the study.

Study Areas	N	MeanEVIi		CvEVII		MeanEVICV		MeanEVIREL		MeanEVIMIN		MeanEVIMAX		rEVIVEC	
		Mean	SE	Mean	SE	Mean	SE	Mean	SE	Mean	SE	Mean	SE	Mean	SE
**JAGUAR**															
Capivara	67	3.537	0.016	0.125	0.003	0.368	0.004	4.009	0.044	1.878	0.019	5.887	0.035	0.794	0.008
Caiman	37	3.899	0.070	0.050	0.002	0.205	0.003	2.946	0.065	2.551	0.047	5.498	0.102	0.778	0.017
Calakmul	23	5.394	0.043	0.048	0.002	0.188	0.002	4.073	0.070	3.441	0.041	7.514	0.073	0.650	0.026
Ducke	24	4.976	0.017	0.056	0.002	0.193	0.003	3.765	0.066	2.902	0.051	6.667	0.043	0.437	0.027
Eden	61	5.719	0.026	0.045	0.001	0.210	0.002	4.796	0.051	3.125	0.026	7.921	0.044	0.542	0.017
Ejido caoba	16	5.603	0.016	0.043	0.001	0.197	0.003	4.811	0.114	3.223	0.059	8.034	0.072	0.585	0.039
Emas	18	3.357	0.076	0.060	0.006	0.244	0.017	3.076	0.172	2.104	0.083	5.180	0.149	0.804	0.020
Maraca	2	5.281	0.102	0.054	0.005	0.184	0.013	4.093	0.538	2.971	0.237	7.065	0.302	0.332	0.068
Petcacab	25	5.590	0.021	0.059	0.002	0.193	0.002	4.668	0.076	3.180	0.051	7.848	0.065	0.671	0.027
Uatuma	7	5.119	0.067	0.073	0.007	0.223	0.005	4.425	0.135	2.524	0.068	6.948	0.097	0.381	0.040
Virua	18	4.261	0.174	0.054	0.003	0.175	0.006	2.940	0.101	2.760	0.138	5.701	0.184	0.338	0.034
Zapotal	64	4.991	0.043	0.069	0.001	0.217	0.002	4.450	0.043	2.783	0.040	7.233	0.063	0.586	0.019
**PUMA**															
Capivara	9	3.489	0.089	0.124	0.008	0.348	0.015	3.831	0.175	1.909	0.059	5.739	0.168	0.795	0.020
Caiman	34	3.844	0.077	0.055	0.003	0.213	0.004	2.994	0.073	2.472	0.053	5.466	0.108	0.799	0.013
Calakmul	27	5.355	0.034	0.052	0.002	0.185	0.002	4.042	0.064	3.390	0.040	7.431	0.058	0.711	0.016
Ducke	64	4.992	0.011	0.054	0.001	0.187	0.002	3.654	0.031	2.930	0.031	6.584	0.025	0.402	0.019
Eden	35	5.533	0.083	0.047	0.003	0.210	0.003	4.704	0.065	3.050	0.058	7.755	0.088	0.562	0.022
Ejido caoba	12	5.597	0.031	0.043	0.003	0.191	0.003	4.587	0.080	3.241	0.066	7.828	0.050	0.584	0.050
Emas	47	3.345	0.052	0.053	0.002	0.219	0.007	2.757	0.065	2.106	0.052	4.863	0.075	0.814	0.011
Maraca	8	5.200	0.046	0.053	0.004	0.176	0.005	3.739	0.126	3.141	0.105	6.880	0.095	0.264	0.067
Petcacab	14	5.595	0.022	0.058	0.002	0.186	0.003	4.431	0.096	3.354	0.050	7.785	0.083	0.661	0.027
Uatuma	12	5.114	0.040	0.071	0.005	0.200	0.006	3.931	0.107	2.973	0.091	6.903	0.069	0.410	0.042
Virua	30	5.179	0.077	0.047	0.002	0.171	0.003	3.572	0.086	3.163	0.059	6.736	0.098	0.464	0.028
Zapotal	55	5.034	0.045	0.063	0.001	0.219	0.003	4.472	0.048	2.790	0.043	7.262	0.060	0.618	0.018
